# Isolating and Validating Fibroblast-like Cells from the Skeletal Muscle of the Siamese Crocodile (*Crocodylus siamensis*)

**DOI:** 10.3390/vetsci13050490

**Published:** 2026-05-19

**Authors:** Nattaphong Akrimajirachoote, Montri Pattarapanawan, Suparat Chaipipat, Yanika Piyasanti, Kornkanok Sritabtim, Juthathip Jurutha, Kannika Siripattarapravat, Piyathip Setthawong

**Affiliations:** 1 Department of Physiology, Faculty of Veterinary Medicine, Kasetsart University, Bangkok 10900, Thailand; nattaphong.a@ku.th; 2 Department of Anatomy, Faculty of Veterinary Medicine, Kasetsart University, Bangkok 10900, Thailand; montri.p@ku.th; 3 Center for Veterinary Diagnostic Laboratory, Bangkhen, Faculty of Veterinary Medicine, Kasetsart University, Bangkok 10900, Thailand; suparat.ch@ku.th (S.C.); yanika.p@kuvacb.com (Y.P.); kornkanok.sr@ku.th (K.S.); juthathip.ju@ku.th (J.J.); kannika.si@ku.th (K.S.); 4 Department of Pathology, Faculty of Veterinary Medicine, Kasetsart University, Bangkok 10900, Thailand

**Keywords:** *Siamese crocodiles*, skeletal muscle, fibroblast isolation, fibroblast-like cells, reptilian cell culture

## Abstract

The isolation of fibroblast-like cells from crocodile skeletal muscle provides a valuable platform for studies in comparative physiology, reptilian biology, regenerative medicine, and tissue engineering. In this article, we present an optimized protocol for isolating and characterizing fibroblast-like cells derived from the embryonic skeletal muscle of the Siamese crocodile (*Crocodylus siamensis*). The procedure improved cell yields and viability while maintaining phenotypic and genetic stability. Dorsal and tail skeletal muscle tissue was cultured in flasks pre-coated with collagen. The cells attached and began migrating from the explants within one day. Optimal culture conditions were achieved using Dulbecco’s Modified Eagle Medium/Nutrient Mixture F-12 or Minimum Essential Medium Alpha supplemented with 10% fetal bovine serum, 2% crocodile serum, and growth-promoting factors, incubated at 28 °C without CO_2_. These conditions supported a shorter population doubling time and enhanced cell proliferation. The established cells displayed a spindle-shaped fibroblastic morphology, expressed the fibroblast-specific marker fibronectin, and maintained a stable karyotype through passage 20. The cell line remained stable and proliferative for at least 30 passages under standard culture conditions. Our study established the first standardized fibroblast-like cell line derived from *C. siamensis*, thus providing a foundation for future studies in reptilian physiology, cytogenetics, and regenerative biotechnology.

## Simple Summary

In this article, we report an optimized protocol for the isolation and characterization of fibroblast-like cells derived from the embryonic skeletal muscle of the Siamese crocodile (*Crocodylus siamensis*). Muscle tissue from the dorsal and tail regions was processed using an explant culture method on collagen-coated flasks, which enabled cell attachment and migration within 24 h. Optimal culture conditions were achieved using Dulbecco’s Modified Eagle Medium/Nutrient Mixture F-12 or Minimum Essential Medium Alpha supplemented with 10% fetal bovine serum, 2% crocodile serum, and growth-promoting factors, with incubation at 28 °C under non-CO_2_ conditions. These conditions significantly enhanced cell proliferation and reduced the population doubling time. The established cells exhibited the characteristic spindle-shaped morphology and expressed fibronectin, thus confirming their fibroblast-like phenotype. Cytogenetic analysis demonstrated a stable diploid karyotype (2n = 30), and the cells maintained proliferative capacity and phenotypic stability for at least 30 passages. To our knowledge, this study represents the first standardized fibroblast-like cell line derived from *C. siamensis* skeletal muscle. The cell line provides a valuable in vitro platform for future studies in comparative physiology, cytogenetics, conservation biology, and regenerative biotechnology.

## 1. Introduction

Cellular models derived from endangered animal species are becoming increasingly important for advancing research in animal physiology while reducing the need for invasive experimentation and supporting ex situ conservation efforts. The Siamese crocodile (*Crocodylus siamensis*) is a critically endangered freshwater crocodile native to Southeast Asia [1]. It is listed under the Convention on International Trade in Endangered Species of Wild Fauna and Flora (CITES) and is classified as critically endangered by the International Union for Conservation of Nature [2]. Wild populations have declined dramatically due to overhunting, habitat loss, and hybridization with saltwater crocodiles, while reduced genetic diversity in captive populations continues to threaten the species’ long-term conservation in the Phetchaburi River and Kaeng Krachan National Park, Thailand [3]. Consequently, the development of biological resources that facilitate conservation, genetic, and physiological studies remains a priority. Beyond its conservation significance, *C. siamensis* has attracted growing biomedical interest due to its remarkable resistance to infection and rapid wound healing. Crocodile-derived bioactive peptides have demonstrated antibacterial, antioxidant, and anti-inflammatory properties [4]. These findings highlight the unique physiological and immune properties of crocodilians and their potential relevance to comparative physiology and regenerative biotechnology [5].

Fibroblasts are mesenchymal cells that play an essential role in extracellular matrix (ECM) production, tissue remodeling, and structural support [6,7]. They are key contributors to wound healing and fibrosis and can differentiate into myofibroblasts in response to mechanical or biochemical stimuli [8]. Although fibroblast biology has been extensively characterized in mammals, including the functional heterogeneity and role of fibroblasts in regeneration and fibrosis, knowledge of fibroblasts derived from reptiles, particularly crocodilians, remains poorly understood [9,10,11,12,13]. Recent studies have demonstrated the feasibility of establishing reptilian and crocodilian fibroblast-like cell cultures; however, these efforts have largely focused on their short-term maintenance or basic cytogenetic assessment, with limited optimization of the culture conditions and insufficient phenotypic characterization [14,15].

Previous studies have demonstrated the feasibility of establishing crocodilian fibroblast-like cell lines. Zeng et al. (2011) [14] successfully established fibroblast-like cell lines from the liver, heart, and skeletal muscle of the Chinese alligator (*Alligator sinensis*) using Dulbecco’s Modified Eagle Medium (DMEM) supplemented with 10% fetal bovine serum (FBS) at 28 °C without CO_2_. The cells, which were subcultured over multiple passages and cryopreserved with 10% dimethyl sulfoxide (DMSO), retained high viability and normal karyotypes after recovery. Similarly, Chumsing et al. (2018) [15] developed a modified primary culture method for *C. siamensis* using eyelid and blood vessel tissue cultured in Iscove’s modified Dulbecco’s medium supplemented with either 10% FBS or 10% crocodile plasma. Fibroblast-like cells, which were observed within two weeks and subcultured up to 10 passages, maintained more than 80% post-thaw viability following cryopreservation. Together, these studies demonstrate the potential of crocodilian fibroblast-like cells as in vitro models for cytogenetic and physiological research, although detailed phenotypic characterization and long-term culture optimization remain limited [5].

Skeletal muscle-derived fibroblasts are important for ECM organization, mechanical support, and muscle regeneration. Establishing an in vitro fibroblast-like cell culture system from *C. siamensis* would expand the availability of reptilian cellular models and provide a practical platform for research in tissue engineering, toxicology, and conservation biotechnology [16]. In addition, muscle-derived fibroblasts are critical supporting cells in muscle culture systems and are increasingly recognized as indispensable components in scaffold-based muscle tissue engineering and cultured meat research [17]. In this study, we aimed to isolate and establish a fibroblast-like cell line from the skeletal muscle of the Siamese crocodile and to optimize culture conditions for its long-term maintenance. We further sought to characterize the established cells through morphological assessment, immunocytochemical analysis, and karyotypic evaluation. This work addresses a critical gap in currently available reptilian cellular models and provides a standardized in vitro platform to support future research in comparative physiology, toxicology, conservation biotechnology, and regenerative biology.

## 2. Materials and Methods

### 2.1. Animal and Ethics Statement

All the procedures in this study were conducted following approval from the Kasetsart University Institutional Animal Care and Use Committee (protocol no. ACKU67-VET-086) and in accordance with national ethical guidelines for animal experimentation.

### 2.2. Tissue Collection and Explant Culture

We obtained embryonic skeletal muscle tissue from a commercial farm (Wongveerakit Farm, Kanchanaburi Province, Thailand). The skeletal muscle tissue was collected from the dorsal and tail regions of *C. siamensis* embryos at 61 days of incubation (*n* = 3). The eggs were opened under sterile conditions, and the embryos were humanely euthanized by rapid decapitation followed by pithing to ensure destruction of the brain tissue in accordance with approved institutional animal care and use guidelines [18]. The yolk contents were then removed, and the dorsal and tail muscle tissue was dissected using sterile scissors and forceps. Two to three grams of the dissected tissue were immediately transferred into sterile phosphate-buffered saline (PBS) supplemented with 1% Antibiotic–Antimycotic (Gibco, Carlsbad, CA, USA) and 2.5 μg/mL amphotericin B (InvivoGen, San Diego, CA, USA) to minimize microbial contamination. The tissue was washed four times with PBS containing antibiotics and then minced into fragments of 1–2 mm^3^ using a sterile surgical blade. For the explant culture, tissue fragments were plated into T25 culture flasks pre-coated with in-house-prepared type I collagen derived from rat tail tendon. Briefly, rat tail tendon collagen was solubilized in 0.1% acetic acid (stock concentration: 4 mg/mL) and stored at −20 °C until use. Prior to coating, the collagen stock solution was diluted 1:1 with sterile distilled water, and 1 mL of the coating solution was added to each flask and incubated at room temperature for 5 h. The flasks were subsequently UV-sterilized for 15 min before the coating solution was aspirated and the explants were seeded. The complete culture medium (3 mL) was then added to the collagen-coated flasks, after which the tissue fragments were gently placed at the bottom of the flasks. The cultures were observed daily using an inverted microscope (Nikon, Tokyo, Japan) to monitor cell migration from the explants and cellular morphology.

### 2.3. Cell Culture and Growth Monitoring

The fibroblast-like cells that migrated from the explants were cultured to determine the optimal growth and maintenance conditions. Two basal media were compared: Dulbecco’s DMEM/Nutrient Mixture F12 (Gibco, Carlsbad, CA, USA) and Minimum Essential Medium Alpha (α-MEM; Gibco, Carlsbad, CA, USA). Each medium was supplemented with 10% FBS (Hyclone, Logan, UT, USA), crocodile serum (0%, 2%, or 5%; collected from adult *C. siamensis*, pooled and heat-inactivated), 1% Antibiotic–Antimycotic, 100 µg/mL Primocin^®^ (InvivoGen, San Diego, CA, USA), 55 µM β-mercaptoethanol (BME; Gibco, Carlsbad, CA, USA), 10 µM p38 inhibitor (MedChemExpress, Monmouth Junction, NJ, USA), and 5 ng/mL basic fibroblast growth factor (bFGF; ImmunoTools, Friesoythe, NI, Germany). The medium pH was adjusted to 7.35–7.40 for DMEM/F12 or 8.00 ± 0.02 for α-MEM, and the solution was subsequently sterilized by filtration. The cultures were maintained in a non-CO_2_ incubator at either 28 °C or 37 °C, and cell morphology, attachment, and proliferation were observed daily using an inverted microscope.

Experimental comparisons to assess cell doubling time were conducted over five passages with three replicates under the following conditions:

1. Basal medium comparison: Cells were seeded at 15,000 cells per well in 24-well plates to compare DMEM/F12 and α-MEM.
2. Crocodile serum supplementation: Cells were seeded at 15,000 cells per well in 24-well plates to compare media supplemented with 0%, 2%, and 5% crocodile serum.
3. Temperature comparison: Cells were seeded at 15,000 cells per well in 24-well plates and cultured at 28 °C or 37 °C.

### 2.4. Subculture of Fibroblast Cells

The primary cultures were monitored daily for cell outgrowth and confluence. When the adherent fibroblast-like cells reached approximately 80% confluency, they were subcultured. The cells were detached using TrypLE^TM^ Select (Thermo Fisher Scientific, Carlsbad, CA, USA) and incubated at 37 °C for 3–5 min until rounded. The cell suspension was gently pipetted to ensure complete dissociation and reseeded at 500,000 cells into new collagen-coated T25 flasks. The culture medium was replaced every 48 h to maintain optimal growth and viability.

### 2.5. Cell Counting and Growth Analysis

The cell proliferation was evaluated at each passage by quantifying the total number of viable cells. The cells were harvested using TrypLE^TM^ Select and resuspended in complete culture medium. Viable cells were counted using a hemocytometer following trypan blue staining to exclude any nonviable cells. The number of cells seeded at the beginning of each passage (*N*_0_) and the number of cells harvested at the end of each passage (*N*_t_) were recorded for subsequent growth analysis.

#### 2.5.1. Cumulative Population Doubling Level

The cumulative population doubling level (CPDL) at each passage was calculated using the following equation:

$$\mathrm{CPDL}=\frac{{log}_{10}(N_{t})-{log}_{10}(N_{0})}{{log}_{10}(2)}$$

where *N*_0_ represents the number of cells initially seeded, and *N*_t_ represents the number of cells harvested at the end of the passage. The CPDL values obtained from each passage were summed to determine the total cumulative population doublings over the entire culture period.

#### 2.5.2. Population Doubling Time

The population doubling time (PDT) was calculated to estimate the average time required for the cells to complete one population doubling. The PDT was determined using the following equation:

$$\mathrm{PDT}=\frac{{log}_{10}(2)\times \Delta t}{{log}_{10}(N_{t})-{log}_{10}(N_{0})}$$

where *N*_0_ represents the number of cells initially seeded, *N*_t_ represents the number of cells harvested at the end of the passage, and Δ*t* represents the culture duration (days) for each passage.

### 2.6. Cryopreservation and Recovery

The cells were harvested and resuspended at approximately 1 × 10^6^ cells/mL in a freezing medium composed of 90% FBS and 10% DMSO (Sigma, St. Louis, MO, USA). The cell suspensions were aliquoted into cryovials (Corning, Jiangsu, China) and placed in a controlled-rate freezing container (Bel-Art Products, Wayne, NJ, USA) with a cooling rate of −1 °C/min at −80 °C overnight. The vials were subsequently transferred to liquid nitrogen (−196 °C) for long-term storage.

For recovery, the cryovials were rapidly thawed in a 37 °C water bath, and the cell suspension was gradually diluted with pre-warmed complete medium. The cells were centrifuged at 800× *g* for 10 min at 25 °C, resuspended in a fresh medium, and seeded into new collagen-coated flasks. Post-thaw cell viability was assessed using a trypan blue exclusion assay.

### 2.7. Immunofluorescence Analysis of Fibroblast Markers

To confirm fibroblast identity, 40,000 cells per well at passage 20 were seeded onto chamber slides (SPL Life Sciences, Pocheon-si, Republic of Korea) and cultured until reaching 60–70% confluence. The cells were fixed with 4% paraformaldehyde (Acros Organics, Fair Lawn, NJ, USA) for 10 min at room temperature, permeabilized with 0.3% Triton X-100 in PBS for 10 min, and blocked with 2% bovine serum albumin (Merck, Darmstadt, Germany) in PBS for 30 min. The primary antibody, anti-fibronectin (Sigma, St. Louis, MO, USA), was diluted 1:100 in PBS and incubated overnight at 4 °C. The secondary antibody, Alexa Fluor^TM^ 568 goat anti-rabbit IgG (1:500, Abcam, Waltham, MA USA), was incubated for 1 h at room temperature. The nuclei were counterstained with Hoechst (1 µg/mL, Sigma, St. Louis, MO, USA) for 10 min. The slides were washed with PBS, mounted using Fluoromount^TM^ (Diagnostic BioSystems, Pleasanton, CA, USA), and visualized under a confocal microscope (Fluoview 3000, Olympus, Tokyo, Japan). Photomicrographs were captured to qualitatively assess marker expression.

### 2.8. Karyotype Analysis

The cells at passage 20 were cultured until they reached 70–80% confluence. The cells were then treated with Colcemid (0.05 µg/mL; Gibco, Carlsbad, CA, USA) for 3 h at 28 °C to arrest the cells in the metaphase. The cells were dissociated into single-cell suspensions and incubated in 0.075 M KCl hypotonic solution (Sigma, St. Louis, MO, USA) for 20 min at 37 °C, followed by fixation in freshly prepared methanol (RCI Labscan, Bangkok, Thailand) and acetic acid (Merck, Darmstadt, Germany) fixative (3:1, *v*/*v*), with the fixative replaced three times. The cell suspensions were dropped onto precleaned slides, air-dried, and stained with 5% Giemsa (Thermo Fisher Scientific, Carlsbad, CA, USA) for 10 min. At least 30 metaphase spreads per passage were imaged using a microscope with a digital imaging system (Nikon, Tokyo, Japan), and chromosome counts were performed.

### 2.9. Statistical Analysis

The data were presented as mean ± standard deviation. Cell doubling time was analyzed according to the experimental design. For the serum concentration experiment, doubling time across passages was analyzed using a two-way repeated-measures analysis of variance, with serum concentration (0%, 2%, and 5%) as the fixed factor and passage number (passages 6–10) as the repeated factor. When significant main effects or interactions were detected, Tukey’s post hoc test was applied for multiple pairwise comparisons among the serum groups.

For the temperature experiment, cells cultured at 37 °C did not survive or proliferate at later passages; therefore, data for those passages were absent. Doubling time was therefore analyzed using a mixed-effects model with restricted maximum likelihood, where temperature (28 °C and 37 °C) was the fixed factor and passage number was the repeated factor. This approach allowed appropriate analysis of the repeated measurements despite the discontinuation of cultures under the 37 °C condition. Statistical significance was set at *p* < 0.05. All statistical analyses were performed using GraphPad Prism version 10.3.1 (GraphPad Software, San Diego, CA, USA).

## 3. Results

### 3.1. Isolation and Culture of Fibroblast-like Cells

**Figure 1:**
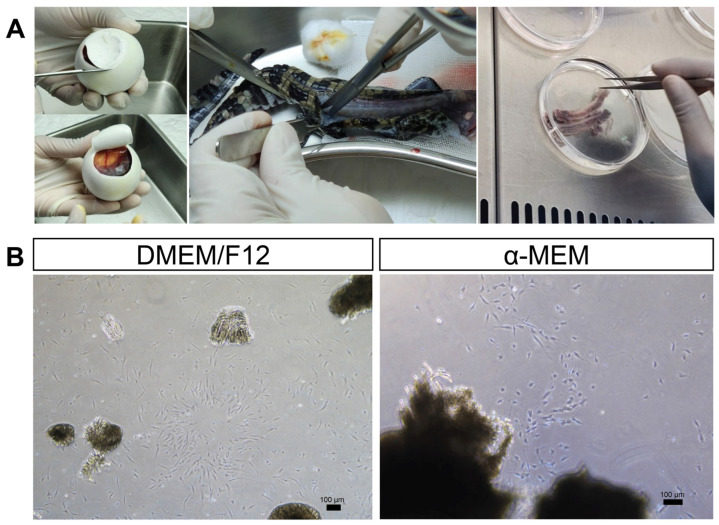
Isolation and primary culture of fibroblast-like cells derived from the embryonic skeletal muscle of the Siamese crocodile. (**A**) Skeletal muscle tissue was aseptically dissected from the dorsal and tail regions of 61-day-old Siamese crocodile embryos and minced prior to explant culture. (**B**) Tissue explants were seeded onto collagen-coated culture surfaces and maintained in Dulbecco’s Modified Eagle Medium (DMEM)/Nutrient Mixture F12 (F12) or Minimum Essential Medium Alpha (α-MEM). Phase-contrast micrographs show fibroblast-like cell outgrowth from the explants on day 4. The outgrowing cells exhibited a characteristic spindle-shaped morphology and demonstrated progressive proliferation over time.

The skeletal muscle tissue obtained from Siamese crocodile embryos at 61 days of incubation yielded a viable population of fibroblast-like cells. The minced muscle tissue was successfully cultured in collagen-coated tissue culture flasks prepared with a 1:1 dilution. Cells adhered firmly to the flask surface and displayed the characteristic spindle-shaped morphology of fibroblasts within one day (Figure 1).

### 3.2. Growth Performance Under Different Culture Conditions

The growth performance of the fibroblast-like cells was assessed over five consecutive passages under different culture conditions. In both DMEM/F12 and α-MEM supplemented with 10% FBS, 1% Antibiotic–Antimycotic, 55 µM BME, 10 µM p38 inhibitor, and 5 ng/mL bFGF, the cells demonstrated sustained proliferative capacity.

**Figure 2:**
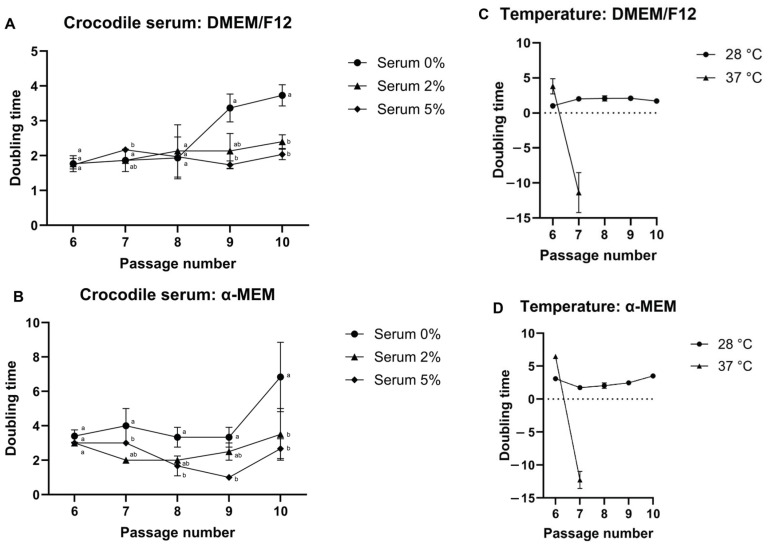
Effects of serum supplementation and temperature on the doubling time of fibroblast-like cells derived from the embryonic skeletal muscle of the Siamese crocodile. The doubling time was evaluated across multiple passages under different culture conditions. (**A**,**B**) Effects of crocodile serum supplementation (0%, 2%, and 5%) in DMEM/F12 and α-MEM, respectively. (**C**,**D**) Effects of incubation temperature (28 °C and 37 °C) in DMEM/F12 and α-MEM, respectively. Cells maintained at 28 °C exhibited stable proliferation with shorter doubling times, whereas those cultured at 37 °C showed impaired growth and could not be maintained through successive passages. Different letters (a, b) indicate significant differences between serum concentrations at the same passage (Tukey’s multiple comparisons test, *p* < 0.05).

The addition of crocodile serum at concentrations of 2% and 5% resulted in comparable cell doubling times, with no statistically significant difference between the two concentrations (Figure 2A,B). Notably, cultures supplemented with either 2% or 5% crocodile serum exhibited significantly shorter doubling times than those without crocodile serum (0%) (*p* < 0.05). Therefore, 2% crocodile serum was selected for subsequent experiments due to its greater cost-effectiveness.

Temperature also significantly influenced cell proliferation. Cells maintained at 28 °C exhibited significantly shorter doubling times than those cultured at 37 °C (Figure 2C,D). Moreover, fibroblast-like cells cultured at 37 °C showed markedly reduced proliferative activity; they failed to proliferate and could not be maintained through successive passages. In contrast, cells cultured at 28 °C retained stable growth and viability across multiple passages, indicating that 28 °C was a more suitable temperature for the long-term culture and maintenance of these cells.

### 3.3. Fibroblast-like Cell Line Maintenance

**Figure 3:**
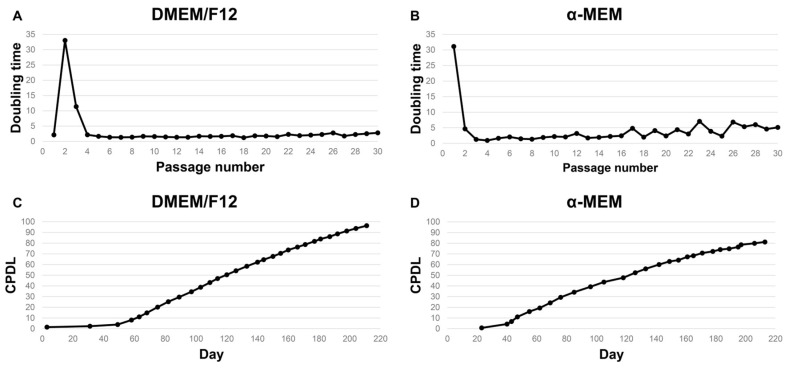
Long-term proliferative capacity of fibroblast-like cells derived from the embryonic skeletal muscle of the Siamese crocodile. The doubling time and cumulative population doubling level (CPDL) were monitored during the serial passaging of two independent back explant-derived cell lines. (**A**,**B**) Doubling time of the fibroblast-like cells cultured in DMEM/F12 and α-MEM, respectively, plotted against passage number during long-term culture. (**C**,**D**) CPDL of cells cultured in DMEM/F12 and α-MEM, respectively. Both cell lines exhibited stabilized growth and sustained proliferation during extended culture, indicating their long-term proliferative capacity.

The fibroblast-like cells reached approximately 80% confluence within 4–6 days and were subsequently subcultured using enzymatic dissociation. The cells were maintained in either DMEM/F12 or α-MEM supplemented with 10% FBS and 2% crocodile serum and cultured at 28 °C. The average cell doubling time was approximately 43 h in DMEM/F12 and 70 h in α-MEM (Figure 3A,B). Routine subculturing every 2–3 days maintained optimal cell density and viability, and the cells consistently exhibited a spindle-shaped morphology when viewed under an inverted microscope. During continuous subculture, the CPDL reached 96 in DMEM/F12 and 81 in α-MEM (Figure 3C,D).

### 3.4. Cryopreservation and Post-Thaw Recovery

**Figure 4:**
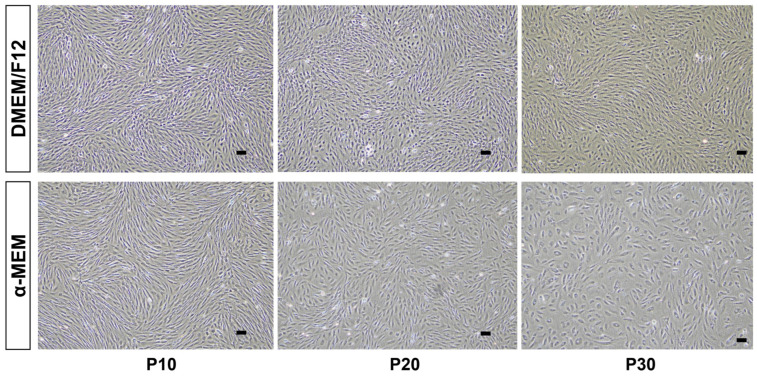
Growth comparison of two fibroblast-like cell lines at passages P10, P20, and P30 cultured in DMEM/F12 or α-MEM under standardized conditions. Growth characteristics were evaluated at each passage to assess any passage-dependent changes in the cell lines. Scale bar: 100 µm.

The fibroblast-like cells were successfully cryopreserved in a freezing medium consisting of 90% FBS and 10% DMSO and stored in liquid nitrogen. Following thawing, cell viability assessed via a trypan blue exclusion assay averaged approximately 90%. The recovered cells rapidly reattached, regained their characteristic spindle-shaped morphology, and resumed active proliferation within 24 h post-thaw. These results indicated that the cryopreservation protocol effectively preserved the viability and proliferative capacity of the cells during long-term storage (Figure 4).

### 3.5. Identification of Fibroblast Cell Markers

**Figure 5:**
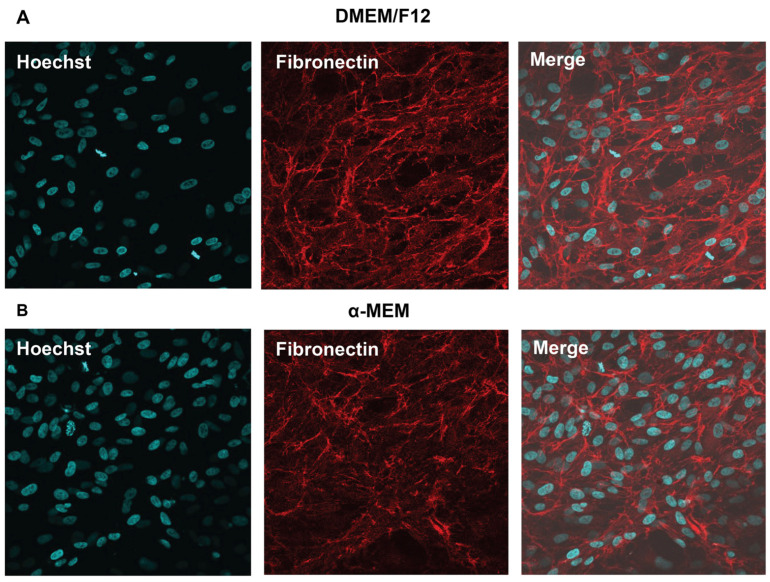
Immunocytochemical identification of fibroblast-like cells derived from the embryonic skeletal muscle of the Siamese crocodile. Cells cultured in (**A**) DMEM/F12 and (**B**) α-MEM were fixed and immunostained for fibronectin, a fibroblast-associated marker. The nuclei were counterstained with Hoechst (blue). Fibronectin staining (red) was predominantly localized in the cytoplasm and ECM, as shown in the single-channel and merged images.

The fibroblast-like identity of the established cell line was confirmed via immunocytochemical analysis. The cells were fixed and stained with an antibody against fibronectin, which is a well-established fibroblast marker. Strong positive staining was predominantly localized in the cytoplasm and ECM, which is consistent with fibronectin expression by fibroblast-like cells. A high proportion of the cells exhibited fibronectin-positive staining, thus supporting the fibroblast-like characteristics and relative homogeneity of the cultured cells (Figure 5).

### 3.6. Karyotype Analysis of Fibroblast-Like Cells

**Figure 6:**
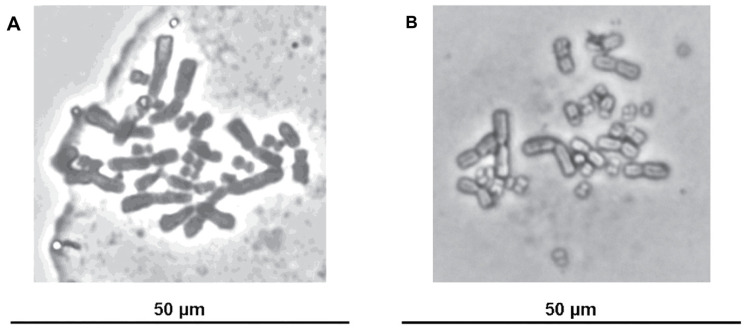
Karyotype of the Siamese crocodile. Representative metaphase chromosome spreads illustrating the diploid chromosome complement (2n = 30). The cells were cultured in (**A**) DMEM/F12 and (**B**) α-MEM media.

Karyotype analysis was successfully conducted on fibroblast-like cells derived from the skeletal muscle of the Siamese crocodile at passage 20. The metaphase chromosome spreads consistently revealed a diploid chromosome number of 2n = 30, which was in agreement with the normal karyotype reported for this species (Figure 6).

## 4. Discussion

In this study, we successfully established and characterized fibroblast-like cells derived from the embryonic skeletal muscle of the Siamese crocodile. An optimized culture protocol for ectothermic reptiles was developed to enable reliable primary culture, long-term maintenance, cryopreservation, and phenotypic validation of crocodilian fibroblast-like cells. This protocol offers a valuable cellular resource for future studies in comparative physiology, cytogenetics, and regenerative biotechnology.

Embryonic muscle tissue was selected because of its higher proliferative capacity and reduced senescence compared with adult-derived fibroblasts [19]. Fibroblast-like cells were efficiently isolated from the embryonic skeletal muscle using an explant culture method supported by a collagen type I coating. This finding was consistent with previous reports showing that collagen supports cell adhesion and migration by providing integrin-binding sites and structural support within the ECM [12]. In both mammalian and reptilian fibroblast cultures, collagen substrates mimic the native ECM environment, allowing cells to retain their spindle-shaped morphology and proliferative capacity [14,15]. The fibroblast-like cells established in this study displayed the typical elongated spindle-shaped morphology and formed layered growth patterns similar to those of mammalian fibroblasts. These findings suggest that key fibroblast characteristics are conserved across vertebrate species [20].

Among the basal media tested, DMEM/F12 supported a shorter PDT and a higher CPDL than α-MEM, indicating enhanced short-term proliferation and a superior long-term proliferative capacity. Because CPDL reflects the cumulative replicative potential of cells across successive passages, it serves as a sensitive indicator of sustained growth performance rather than transient proliferative responses [21,22]. The higher CPDL observed with DMEM/F12 indicates that this medium preserved the replicative capacity of the cells more effectively during the extended culture, likely due to its enriched nutrient formulation and superior buffering capacity. In contrast to α-MEM, DMEM/F12 contains a higher glucose concentration, a broader range of amino acids and vitamins, and better buffering capacity, which together support enhanced cellular metabolism and sustained proliferation [23]. These observations align with previous reports indicating that basal medium composition plays a critical role in long-term cell expansion. For example, DMEM/F12 has been shown to enhance osteogenic differentiation and proliferative performance in human mesenchymal stem cells [24]. Similarly, Pathak et al. (2023) reported that adult caprine dermal fibroblasts cultured in DMEM/F12 exhibited faster proliferation, higher colony-forming efficiency, and enhanced migratory capacity compared with those cultured in α-MEM [25]. The suitability of DMEM-based media for fibroblast maintenance has also been observed in reptilian models, including *Alligator sinensis*, in which fibroblast cultures showed improved growth under DMEM-based, non-CO_2_ conditions [14].

Temperature also had a significant impact on fibroblast proliferation in this study, with cells exhibiting more rapid growth at 28 °C than at 37 °C. This finding is consistent with previous reports of temperature-dependent growth in reptilian cells, in which optimal viral replication and host–cell metabolism in snake kidney cells occurred between 28 °C and 32 °C [26]. Similarly, primary dermal fibroblast cultures from the olive ridley sea turtle (*Lepidochelys olivacea*) were maintained at 26 °C under 5% CO_2_ conditions, further supporting the use of lower incubation temperatures for reptilian cell culture systems [27]. These results emphasize that ectothermic cells function more efficiently at lower temperatures that reflect their natural physiological range rather than mammalian body temperatures [28].

Reptiles are poikilothermic (ectothermic) organisms whose physiological processes are strongly influenced by environmental temperatures [29,30]. Unlike endothermic species, reptiles rely on ambient conditions to regulate cellular metabolism, enzyme activity, and overall biological function. Consequently, the species-specific Preferred Optimal Temperature Zone (POTZ) is important for maintaining normal physiological performance. In vitro, culture conditions approximating the POTZ are therefore likely to improve cell viability, proliferation, and functional stability. In the present study, an incubation temperature of 28 °C was selected based on the known thermal biology of *C. siamensis* and is consistent with temperatures reported for other reptilian cell culture systems [31]. Compared with conventional mammalian culture conditions, this lower temperature likely contributed to the observed cell stability and proliferation by reflecting the metabolic characteristics of ectothermic organisms. The successful maintenance of crocodilian fibroblast-like cells at 28 °C under non-CO_2_ conditions further suggests that specialized mammalian-style incubation conditions may not be required for reptilian cell culture [15]. This simplified culture approach may also reduce operational complexity and cost.

Supplementation with crocodile serum significantly enhanced fibroblast proliferation, with concentrations of 2–5% producing the most favorable effects. This response is likely attributable to the species-specific bioactive components present in crocodilian serum, including antioxidant and cell-modulating factors previously shown to promote wound healing and cellular migration [32,33]. In addition, the inclusion of a p38 MAPK inhibitor may have contributed to enhanced cell expansion by suppressing stress-induced senescence during primary cell establishment under low-temperature, non-CO_2_ culture conditions [34,35]. Together, these findings underscore the importance of optimizing species-specific supplementation and targeted signaling modulation to maintain reptilian cell viability and sustained proliferative capacity in vitro.

The fibroblast-like identity of the cell line established in our study was confirmed by the cytoplasmic expression of fibronectin, a hallmark ECM glycoprotein, with uniform positivity indicating a high degree of cellular homogeneity [36]. Fibronectin expression and the characteristic fibroblast morphology were maintained through at least 20 passages, demonstrating high phenotypic stability [37]. Chromosomal integrity was verified by karyotyping, which revealed a normal diploid complement (2n = 30) consistent with *C. siamensis*, thereby supporting the utility of this cell line for cytogenetic analyses and conservation biology [38]. Cryopreservation resulted in approximately 90% post-thaw cell viability, with preserved cellular morphology and marker expression, supporting the prospect of long-term cell banking and highlighting its potential for use in future assisted reproduction technologies and as a genetic resource for endangered reptile conservation [39,40].

The successful derivation and long-term maintenance of fibroblast-like cells from *C. siamensis* provide a controllable in vitro platform for investigating crocodilian biology, including wound repair and innate immune responses, as previously demonstrated in studies of crocodile plasma- and serum-mediated tissue regeneration [33]. These fibroblasts may also secrete species-specific ECM components or antimicrobial peptides, highlighting their potential utility in biomaterials development and pharmaceutical applications [41]. Importantly, muscle-derived crocodilian fibroblasts can contribute to scaffold remodeling and provide essential structural and biochemical cues within tissue-engineered constructs, thereby supporting cell organization and maturation in engineered muscle systems [42,43]. Although fibroblasts alone do not form muscle tissue, their inclusion is critical for the development of complex three-dimensional constructs, including cultured meat platforms and organoid systems, where they support tissue architecture and functional integration [44].

Establishing a stable, species-specific fibroblast line is a critical step toward reptilian cell-based tissue engineering and conservation biotechnology. However, the present study has limitations that warrant consideration. First, although the established cells exhibited fibroblast-like morphology and fibronectin expression, additional characterization using a broader panel of positive fibroblast markers (e.g., vimentin, collagen I, and FSP1/S100A4) together with negative lineage markers (e.g., desmin, MyoD, myogenin, or epithelial markers) would further strengthen the confirmation of cell identity and help exclude potential contamination from myogenic or epithelial cell populations [45]. The absence of these markers partly reflects the limited availability and validation of species-specific antibodies for reptilian models [46]. Future studies should therefore incorporate broader multi-marker validation approaches, including transcriptomic or proteomic profiling, to achieve more definitive cellular characterization.

Second, the use of embryonic skeletal muscle as the source tissue may limit physiological relevance when extrapolating findings to adult organisms, as embryonic cells typically exhibit higher proliferative capacity and distinct molecular profiles. Comparative studies using fibroblast-like cells derived from adult tissues and across developmental stages will therefore be important to better relate these in vitro findings to in vivo biology [47]. In addition, fibroblasts derived from other crocodilian tissues, such as the dermis, liver, and heart, should be investigated to enable comparisons of their physiological and genomic characteristics [48]. Such cross-tissue analyses may reveal lineage-specific features and provide deeper insights into crocodilian regeneration, immune function, and stress adaptation [6,9]. Furthermore, integrating these cell culture models with genomic and proteomic approaches may facilitate the identification of novel molecules underlying crocodilian resilience with potential biomedical applications.

## 5. Conclusions

In this study, we successfully established and optimized fibroblast-like cell cultures from *C. siamensis.* A collagen coating, DMEM/F12 medium, supplementation with 2% crocodile serum, and incubation at 28 °C were identified as key conditions supporting robust proliferation and long-term stability of these cultures. The cultured cells maintained characteristic fibroblast morphology and normal chromosomal integrity across multiple passages and exhibited high post-thaw viability following cryopreservation. Collectively, these findings provide a reliable in vitro platform and valuable cellular resource for advancing studies in reptilian physiology, tissue engineering, and conservation biotechnology.

## Data Availability

The original contributions presented in this study are included in the article. Further inquiries can be directed to the corresponding author.
